# Differential modulation of neural oscillations in perception-action links in Tourette syndrome

**DOI:** 10.1093/braincomms/fcaf172

**Published:** 2025-05-05

**Authors:** Astrid Prochnow, Annet Bluschke, Tina Rawish, Julia Friedrich, Yifan Hao, Christian Frings, Tobias Bäumer, Alexander Münchau, Christian Beste

**Affiliations:** Cognitive Neurophysiology, Department of Child and Adolescent Psychiatry, Faculty of Medicine, TU Dresden, 01307 Dresden, Germany; Cognitive Neurophysiology, Department of Child and Adolescent Psychiatry, Faculty of Medicine, TU Dresden, 01307 Dresden, Germany; Institute of Systems Motor Science, University of Lübeck, 23562 Lübeck, Germany; Cognitive Neurophysiology, Department of Child and Adolescent Psychiatry, Faculty of Medicine, TU Dresden, 01307 Dresden, Germany; Institute of Systems Motor Science, University of Lübeck, 23562 Lübeck, Germany; Cognitive Psychology, University of Trier, 54286 Trier, Germany; Institute of Systems Motor Science, University of Lübeck, 23562 Lübeck, Germany; Institute of Systems Motor Science, University of Lübeck, 23562 Lübeck, Germany; Cognitive Neurophysiology, Department of Child and Adolescent Psychiatry, Faculty of Medicine, TU Dresden, 01307 Dresden, Germany; German Center for Child and Adolescent Health (DZKJ), partner site Leipzig/Dresden, 01307 Dresden, Germany

**Keywords:** Tourette syndrome, response inhibition, theta band activity, alpha band activity, perception–action integration

## Abstract

Gilles de la Tourette Syndrome (GTS) is a multi-faceted neuro-psychiatric disorder. While novel conceptions overcoming the criticized categorization of GTS as a movement disorder are on the rise, little is known about their neural implementation and whether there are links to known pathophysiological processes in GTS. This is the case for conceptions suggesting that aberrant perception–action processes reflect a key feature of GTS. Building on the concept that overly strong perception–action associations are pivotal to understanding GTS pathophysiology, we examined how these associations influence response inhibition and used EEG methods to examine the importance of theta, alpha and beta band activity due to their known relevance for GTS pathophysiology. In this case–control study, behavioural analyses revealed that adult patients with GTS experienced greater difficulty during motor response inhibition when perceptual features of Nogo stimuli overlapped with perceptual features of Go stimuli, indicating impaired reconfiguration of perception–action associations. Neurophysiological findings showed robust differential patterns of modulation in theta and alpha band activity between neurotypical (NT) individuals and GTS patients. Specifically, GTS patients exhibited stronger and more extended theta band modulation but weaker and more restricted alpha band modulation during overlapping Nogo trials than NT individuals. Unlike NT individuals, GTS patients did not exhibit beta band modulations necessary for dynamically handling perception–action codes. The findings highlight increased theta band modulation in GTS patients’ significant stronger perception–action bindings and a lack of compensatory alpha band modulation. The robust differential modulation observed provides novel insights, emphasizing theta and alpha oscillations as key elements in GTS pathophysiology and offering potential implications for targeted cognitive-behavioural interventions.

## Teaser

Gilles de la Tourette Syndrome (GTS) is linked to altered perception–action associations. In an EEG study, GTS patients showed greater response inhibition difficulties when restructuring these associations. Stronger theta but weaker alpha modulation suggests overly rigid perception–action bindings with insufficient compensatory processes, highlighting oscillatory alterations underlying GTS pathophysiology.

## Graphical Abstract

**Graphical Abstract:**
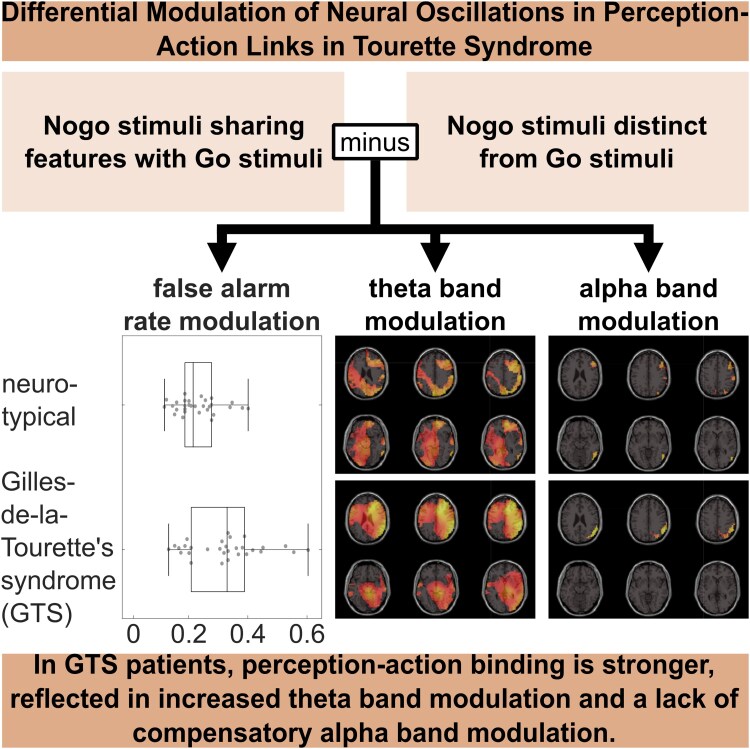

## Introduction

Gilles de la Tourette Syndrome (GTS) is a multi-faceted neuro-psychiatric disorder with tics as the defining feature.^1,2^ It has long been referred to as a movement disorder,^3^ but this view is increasingly questioned.^3-5^ In recent years, there has been an increasing interest in novel conceptual views that can frame the phenomenology of GTS. One of these views suggests that GTS may be conceptualized as a condition characterized by too tight associations or bindings between perceptual and motor processes.^3^ Due to these too tight bindings, increased efforts are necessary to reconfigure these associations in the face of changing relationships of stimuli and responses.^3^ Several lines of evidence have corroborated this novel conceptual view of GTS being a disorder of overly strong integration of perceptual and motor processes.^6-10^ Importantly, these data may also be relevant to better understanding the mechanisms of behavioural interventions to treat GTS symptoms.^11^ Yet, the neural underpinnings of this new GTS phenomenology conception are currently unclear, and whether such neural processes are relevant to known pathophysiological aspects in GTS is elusive. This is particularly the case with respect to neural oscillatory activity central for information processing in the brain^12^ and novel concepts regarding the role of activity in theta, alpha and beta frequency bands during perception–action integration in fronto-parietal circuits.^13^ According to these recent conceptions,^13^ theta band activity likely reflects ongoing integration (binding) and retrieval processes of newly formed perception–action associations, alpha band activity likely modulates/controls these theta band-associated processes, and beta band activity predominantly serves the storage of perception–action associations. These conceptions provide possible important links to the pathophysiology of GTS:

There is increasing evidence that activity in the theta frequency band in fronto-striatal networks is particularly important in the pathophysiology of GTS^14-16^ and also relevant for its clinical phenotype.^17,18^ Given the prominent role of theta band activity in GTS pathophysiology and perception–action integration, we hypothesize that particularly theta band activity modulations reflect the problems of individuals with GTS to adapt action control processes based on altered sensory input, compared to neurotypical individuals. If so, this will have profound implications for the understanding of GTS since it will allow us to link a novel concept regarding the importance of perception–action integration processes with pathophysiological alterations related to the occurrence of tics.

Given its role in tic control, alpha band activity is also relevant. Alpha band activity is important in GTS's pathophysiology when controlling tics.^19,20^ Moreover, as shown recently, it likely affects theta band-associated perception–action integration processes.^13,21,22^ If an exaggerated theta band activity in GTS gives rise to overly strong perception–action associations, the question is how alpha band activity is modulated. No clear a priori hypotheses can be formulated regarding the modulation of alpha band activity between conditions requiring to adapt action control processes based on changed sensory input and those that don't in GTS, compared to neurotypical individuals. If alpha band activity is modulated differently than theta band activity in individuals with GTS and also explains alterations in perception–action integration, this would broaden the significance of alpha band activity for the understanding of the pathophysiology of GTS. Finally, investigating beta band activity is important since it is likely key for storing perception–action associations.^13^ There is also evidence suggesting that changes in beta band activity in fronto-striatal structures are pathophysiologically relevant in GTS,^23-27^ potentially indicating an overactivation of motor processes during movement.^28,29^ Of note, alterations in the modulation of oscillatory activity in GTS may occur in terms of either the magnitude, the spatial extent, or both. Therefore, in addition to comparing the magnitude of activity modulation between groups, the size of activity modulation clusters in the brain should also be examined.

To examine the questions on the possible relevance of different oscillatory activity dynamics for perception–action integration processes in GTS, we here focus on perception–action integration during response inhibition processes in an EEG-beamforming study. This increases the relevance of this study because response inhibition processes have long been discussed to be altered in GTS, with results being conflicting.^9,30-37^ We use an established Go/Nogo task, in which perceptual overlaps between Go and Nogo stimuli can complicate deciding whether to execute or inhibit a response.^38^ To allow response inhibition, perception–action codes established during frequent Go trials must be reconfigured in some Nogo trials, hampering successful inhibitory control. However, there is no necessity in other instances. It is hypothesized that patients with GTS experience difficulties in the reconfiguration process compared to neurotypical individuals, and that these alterations are reflected in the neurophysiological activity underlying this reconfiguration process.

## Materials and methods

### Sample

The sample consisted of a group of *N* = 30 adult participants diagnosed with GTS (mean age 26.6 ± 6.7 years, age range 19–40 years, 18 male) and a group of *N* = 30 adult participants without any psychiatric diagnosis (neurotypical individuals; NT) (mean age 25.6 ± 4.1 years, age range 19–37 years, 11 male) who were matched for age and sex. This sample size is comparable to previous studies examining tics and GTS in adults,^39-41^ and for the current study design (mixed-effects ANOVA with two groups and two conditions) allows to detect small to medium effects (*f* = 0.237) with a power of 95%.^42^ Diagnostic confirmation was performed at the specialized GTS outpatient clinic at the University Medical Center Schleswig-Holstein, Campus Lübeck, Germany. Recruitment of controls was carried out through announcements distributed via the university's email network. The groups did not differ significantly with respect to age (*t*(48.3) = 0.72, *P* = 0.476) and gender distribution (χ^2^(1, *N* = 60) = 3.27, *P* = 0.071).

All participants underwent a standardized assessment protocol, which included a structured clinical interview (Mini-DIPS^43^), and evaluations of obsessive-compulsive symptoms (Yale-Brown Obsessive Compulsive Scale, Y-BOCS^44^). In the patient group, additional assessments included measures of tic severity (Yale Global Tic Severity Scale, YGTSS^45^; Diagnostic Confidence Index for GTS, GTS-DCI^46^). The data collection took place between January 2022 and August 2024.

Among the NT individuals, Mini-DIPS and Y-BOCS assessments showed no evidence of current or past clinically relevant psychiatric disorders. Four participants reported occasional cannabis use, and one of them also reported occasional MDMA use.

In the GTS group, *N* = 4 participants had a prior diagnosis of ADHD. A history of depressive episodes was identified in *N* = 7, and symptoms consistent with current or past anxiety disorders were observed in *N* = 8. These included panic disorder (*N* = 4), social anxiety disorder (*N* = 6), specific phobia (*N* = 3) and generalized anxiety disorder (*N* = 2). Clinically relevant obsessive-compulsive symptoms, indicated by a Y-BOCS score exceeding 16, were found in *N* = 3 participants. At the time of study participation, *N* = 11 patients were receiving pharmacological treatment for tic management: aripiprazole (*N* = 5), nabiximols (*N* = 1), aripiprazole combined with nabiximols (*N* = 1), Bedrocan (*N* = 1), sulpiride (*N* = 1), amisulpride (*N* = 1) and pimozide (*N* = 1). Patients with GTS reported an average symptom duration of 19.0 ± 7.3 years. On the YGTSS, the mean motor tic severity score was 14.5 ± 3.8, vocal tic severity score was 8.1 ± 6.3, and overall tic severity score was 41.9 ± 15.9. The GTS-DCI showed a mean diagnostic confidence score of 63.5 ± 18.5. The individual demographic and clinical characteristics of the patients with GTS are presented in Supplementary Table 1. Prior to participation, all subjects provided written informed consent. The study was conducted in compliance with the Declaration of Helsinki (1964) and received approval from local ethics committees.

### Task

**Figure 1:**
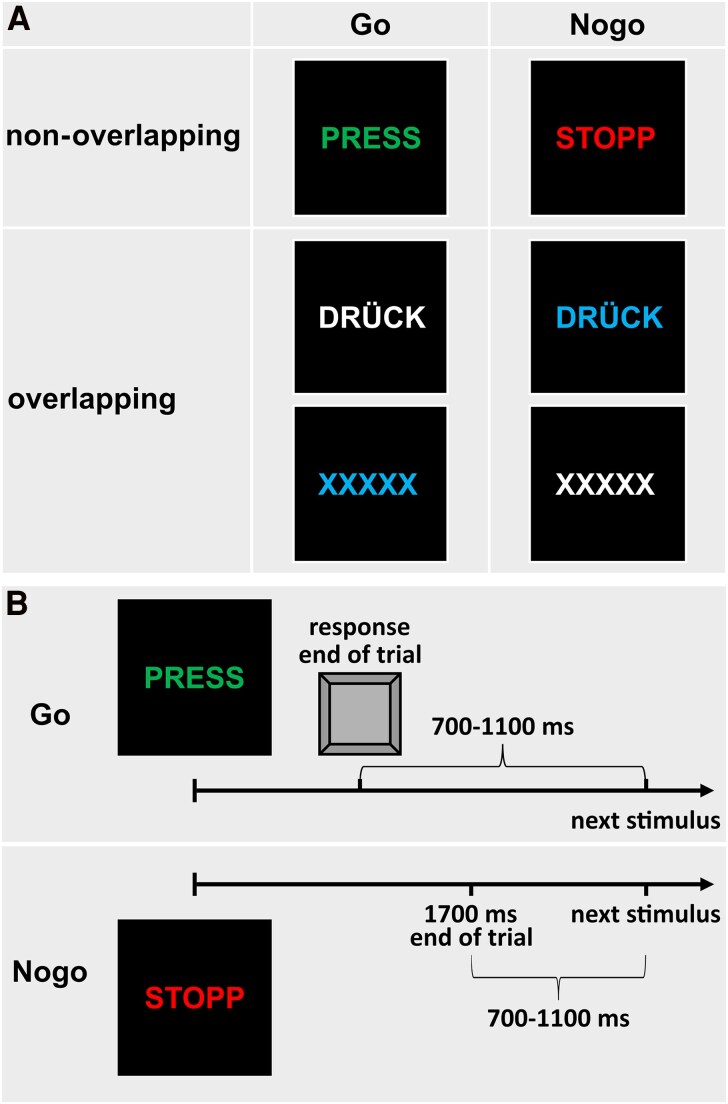
**Depiction of task stimuli**. (**A**) In the non-overlapping condition, the Go and Nogo stimuli differed in both features (i.e. letters and font colour), while in the overlapping condition, Go and Nogo stimuli shared similar features. (**B**) A trial either ends either with a key press or after 1700 ms. For illustration, a correct non-overlapping Go trial and a correct non-overlapping Nogo trial are presented.

To examine the integration of perception and action in response inhibition, GTS patients and NT participants completed a Go/Nogo task where perception–action codes must be reconfigured in some instances, while there is no necessity to do so in other instances.^47,48^ This task included Go trials, which required a response, and Nogo trials, requiring response inhibition. Some trials featured overlapping characteristics (such as shared colour or letter properties) across Go and Nogo conditions, while others did not share features. The stimuli used in the task and the procedure of a typical trial are displayed in Fig. 1.

Overlapping features created partially linked event files, as the shared properties were associated with both responses and inhibitions, likely increasing the false alarm rate on Nogo trials. In contrast, non-overlapping trials had distinct features for each condition: non-overlapping Go trials displayed the green word ‘PRESS’ while non-overlapping Nogo trials showed the red word ‘STOPP.’ Two types of overlapping Go and Nogo trials were presented. In the first overlapping Go condition, the word ‘DRÜCK’ (meaning ‘press’ in German) appeared in white, while in the second, the letters ‘XXXXX’ appeared in blue. For overlapping Nogo trials, either ‘DRÜCK’ appeared in blue or ‘XXXXX’ in white. Participants responded to Go trials by pressing the space bar with their right index finger as quickly as possible. A practice session was conducted to familiarize participants with the task before the actual test.

The main task included 196 Go trials and 84 Nogo trials for both overlapping and non-overlapping conditions, maintaining a 70:30 Go-to-Nogo ratio to encourage a strong response tendency. The task was divided into seven equally sized blocks, with trials presented in a pseudorandomized order. Participants controlled the break duration between blocks. Each stimulus appeared for 450 ms, with trials lasting until a response was made or until 1700 ms had elapsed without a response. The time between trials varied randomly from 700 to 1100 ms, with a fixation cross displayed at the screen centre when no stimulus was present.

For analysis, overlapping Go and Nogo trials were grouped separately, as were non-overlapping trials. The false alarm rate for Nogo trials and reaction times for Go trials were averaged for each feature overlap type. Only Nogo trials were used in neurophysiological analysis to focus on event file dynamics related to inhibitory control. The ‘overlap effect’ was calculated as the difference between the overlapping minus the non-overlapping condition.

### EEG recording and pre-processing

EEG data were recorded with a BrainAmp Amplifier and Brain Vision Recorder 1.2 software (Brain Products, Germany) using 60 Ag/AgCl electrodes arranged in an equidistant layout and sampled at 500 Hz. The ground electrode was positioned at θ = 58, ϕ = 78, and the reference electrode at θ = 90, ϕ = 90. Off-line pre-processing was performed with the Brain Vision Analyzer 2 software (Brain Products Inc., Germany). First, the data were re-sampled to 256 Hz, and an 8th-order IIR bandpass filter (0.5–40 Hz) and a 50 Hz notch filter were applied. Non-responsive channels were removed, and the data were re-referenced to an average reference. During the manual inspection, technical artefacts were removed, followed by an independent component analysis (ICA, Infomax) to eliminate periodic artefacts. Any remaining artefacts were removed during a second review of the raw data, after which previously discarded channels were interpolated topographically. For all conditions, individual segments were created around each trial's stimulus (−2000 to 2000 ms). An automatic artefact rejection then removed segments with amplitudes over 200 µV or below −200 µV or with activity below 0.5 µV for more than 100 ms. Finally, baseline correction was performed based on the mean activity in the −200 to 0 ms window before stimulus onset.

### Time–frequency decomposition

Time–frequency analysis was conducted using the FieldTrip toolbox for MATLAB^49^ to examine oscillatory power dynamics. Specifically, wavelet-based time–frequency decomposition was performed to obtain power values. To ensure adequate frequency resolution, zero-padding was applied, with the padding length calculated as the ceiling of the maximum trial length (in samples) divided by the sampling rate. The frequencies of interest ranged from 3 to 30 Hz. The analysis included all time points across the trial duration. For each group and condition, grand averages were calculated.

Subsequently, data were compared within the theta (4–7 Hz), alpha (8–12 Hz) and beta (15–30 Hz) frequency bands using cluster-based permutation testing, as implemented in FieldTrip. For within-group comparisons, cluster-based permutation tests were conducted to assess differences between the non-overlapping and overlapping Nogo conditions. The analysis used Monte Carlo simulations with 1000 randomizations to evaluate statistical significance at an alpha level 0.05. The data were averaged across time points and frequencies within each frequency band of interest. A two-tailed design with correction for multiple comparisons via clustering was implemented, and a dependent-sample *t*-test was used to calculate within-group contrasts. Between-group comparisons were performed on the two Nogo conditions and the overlap effect (overlapping Nogo minus non-overlapping Nogo). Similarly, this time, cluster-based permutation tests were conducted using an independent-samples *t*-test. For both within- and between-group comparisons, electrode neighbourhood information was defined to enable spatial clustering, with a minimum of two neighbouring channels required per cluster. All configuration options left unspecified in the code were set to FieldTrip's default values. An analysis of the relationship of alpha and theta band activity can be found in the Supplemental Material.

### Beamforming

Electrode configurations were aligned with a standard 61-channel EEG montage and a boundary element method head model. Data were restricted to a time window of interest (0–1 s) for both Nogo conditions before being appended to create a common spatial filter. For each frequency band of interest, cross-spectral density (CSD) matrices were computed for each condition and their combination using multitaper frequency analysis with Hanning tapering. For each frequency band, a common spatial filter was constructed via Dynamic Imaging of Coherent Sources (DICS) beamforming,^50^ applying a rank reduction of 3% and 5% lambda regularization. Using the common spatial filter, source-level power was estimated for each condition.

Contrasts between conditions were computed directly at the source level using relative power differences, i.e. normalizing the difference between the power in the Nogo conditions by the sum of the power in both Nogo conditions. Grand averages were computed across participants within each group for each combination of condition and frequency bands. Cluster-based permutation testing was applied using the FieldTrip toolbox to identify significant differences between the Nogo conditions within each group. The statistical analysis used a dependent-samples *t*-test for each voxel, and the cluster-based permutation procedure was employed to correct for multiple comparisons. A Monte Carlo approach generated a null distribution of the test statistic with 1000 randomizations. Clusters with a *P* < 0.05 were considered significant. All unspecified parameters were set to FieldTrip's defaults.

To identify brain regions showing the largest difference between the two Nogo conditions, Density-Based Spatial Clustering of Applications with Noise (DBSCAN)^51^ was applied to the source-level contrasts. Non-labelled and cerebellar structures were excluded based on the anatomical region. Subsequently, the data were thresholded based on the top 1% power difference values. The minimum voxel size for a cluster was set at 5 voxels, and the search radius was set at 1.5 times the grid size of 5 mm. Clusters were labelled based on the Automatic Anatomical Labeling (AAL) atlas.^52^

### Statistical analysis

For the analysis of the behavioural data, Go hit rates, Go reaction times and Nogo false alarm rates were analysed using mixed-effects ANOVAs with the within-subjects factor ‘overlap’ (non-overlapping versus. overlapping) and the between-subjects factor ‘group’ (GTS versus. NT). To further examine group differences, *post hoc* tests were conducted. Prior to *post hoc* testing, Shapiro–Wilk tests were employed separately for each group to assess the normality of the dependent variables. If the data were normally distributed, dependent and independent *t*-tests were calculated, respectively. If the data were not normally distributed, non-parametric Wilcoxon and Mann–Whitney U-tests were computed, respectively. Similarly, overlap effects were compared between groups. Of note, as the main interpretation will follow the *post hoc* tests, a violation of the normality assumption for the ANOVA would be acceptable.^53^

To enable a statistical evaluation of the results of cluster-based permutation testing between both groups, the cluster-based permutation testing was conducted again using a leave-one-out cross-validation approach, where one subject was excluded in each iteration to evaluate the robustness of the results. The cluster statistics (*T*_sum_) and the number of significant voxels were recorded for each identified cluster. Subsequently, we analysed the results from cluster-based permutation testing using non-parametric statistical methods. First, we calculated a relative *T*_sum_ by dividing the total *T*_sum_ by the number of significant voxels for each frequency band (theta, alpha, beta). For the first comparison approach, missing values were assigned when no significant clusters were identified in the cluster-based permutation test. In a second, more conservative approach, the absence of significant clusters was ‘penalized’ by assigning them a zero value. Non-parametric Mann–Whitney U-tests were conducted for each frequency band to compare the differences between the groups.

To examine the relationships among behavioural effects, neurophysiological effects, and, in the patient group, clinical measurements, correlational analyses were conducted. Given that the neurophysiological results were derived using a leave-one-out approach for each group, a similar method was applied to the behavioural results and clinical measurements. Specifically, averages were calculated excluding each subject in turn to ensure consistency and comparability across variables. Due to the distributional changes introduced by this leave-one-out approach, Spearman's rank correlation coefficient (*ρ*) was used to assess monotonic relationships. To account for the multiple comparisons performed (48 correlations of interest; see Supplementary Table 2), the significance threshold (*α*) was Bonferroni-corrected and set at 0.001.

## Results

### Behavioural results

**Figure 2:**
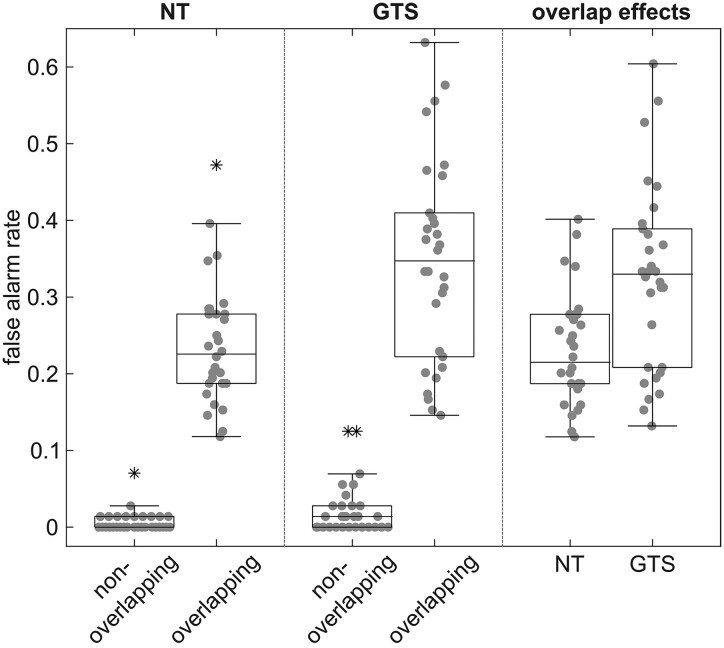
**Behavioural data**. Boxplots of false alarm rates in Nogo trials in non-overlapping and overlapping trials in neurotypical individuals (NT; *N* = 30) and patients with GTS (*N* = 30), along with the overlap effect in both groups. Boxes represent the interquartile range (IQR) from the first quartile (Q1) to the third quartile (Q3), with the median indicated by a horizontal line. Whiskers extend to 1.5 × IQR from Q1 and Q3. Individual data points are shown as grey dots, and outliers are marked with asterisks. The mixed-effects ANOVA of the false alarm rates in Nogo trials revealed an interaction of Overlap ∗ Group (*F*(1,58) = 12.93, *P* < 0.001), as the overlap effect was larger in patients with GTS than in NT (*t*(47.46) = 3.60, *P* < 0.001).

The behavioural data are displayed in Fig. 2.

Shapiro–Wilk tests indicated that Go hit rates were not normally distributed in either the non-overlapping or overlapping conditions across both groups (*W* ≤ 0.77, *P* < 0.001). With respect to the mixed-effects ANOVA of the Go hit rates, only a significant main effect of the factor Overlap was established (*F*(1,58) = 18.54, *P* < 0.001, η^2^*_p_* = 0.242), with higher hit rates in non-overlapping (0.98 ± 0.03) compared to overlapping trials (0.97 ± 0.04). Effects encompassing the factor Group did not reach significance (*F* ≤ 2.40, *P* ≥ 0.127).

With respect to Go reaction times, Shapiro–Wilk tests indicated that normality was only present in the NT group (*W* ≥ 0.93, *P* ≥ 0.058), but not in the GTS group (*W* ≤ 0.91, *P* ≤ 0.015). Regarding the mixed-effects ANOVA of the Go reaction times, only a significant main effect of the factor Overlap was revealed (*F*(1,58) = 7.61, *P* = 0.008, η^2^*_p_* = 0.116), with faster reaction times in non-overlapping (490 ± 46 ms) compared to overlapping trials (498 ± 49 ms). Effects encompassing the factor Group did not reach significance (*F* ≤ 1.52, *P* ≥ 0.222).

For the Nogo false alarm rates, Shapiro–Wilk tests indicated that values were not normally distributed in the non-overlapping condition (*W* ≤ 0.70, *P* < 0.001) but in the overlapping condition (*W* ≥ 0.96, *P* ≥ 0.069). The mixed-effects ANOVA of the false alarm rates in Nogo trials revealed significant main effects of the factors Overlap (*F*(1,58) = 462.87, *P* < 0.001, η^2^*_p_* = 0.889) and Group (*F*(1,58) = 14.67, *P* < 0.001, η^2^*_p_* = 0.202). False alarm rates were higher in overlapping (0.29 ± 0.12) compared to non-overlapping trials (0.02 ± 0.03), as well as in patients with GTS (0.18 ± 0.08) compared to NT (0.12 ± 0.04). Most importantly, also the interaction of Overlap ∗ Group (*F*(1,58) = 12.93, *P* < 0.001, η^2^*_p_* = 0.182) was significant. The groups differed with respect to the non-overlapping (GTS: 0.02 ± 0.03; NT: 0.01 ± 0.01; *Z* = −2.08, *P* = 0.038, *r* = −0.379) as well as the overlapping condition (GTS: 0.35 ± 0.13; NT: 0.24 ± 0.08; *t*(47.7) = 3.81, *P* < 0.001, *d* = 0.982), while both groups showed a significant overlap effect (GTS: *Z* = −4.78, *P* < 0.001, *r* = −0.873; NT: *Z* = −4.79, *P* < 0.001, *r* = 0.874). However, the overlap effect was larger in patients with GTS (0.32 ± 0.12; *W* = 0.96, *P* = 0.345) than in NT (0.23 ± 0.07; *W* = 0.95, *P* = 0.158; *t*(47.46) = 3.60, *P* < 0.001, *d* = 0.929).

### Cluster-based permutation testing on sensor level

**Figure 3:**
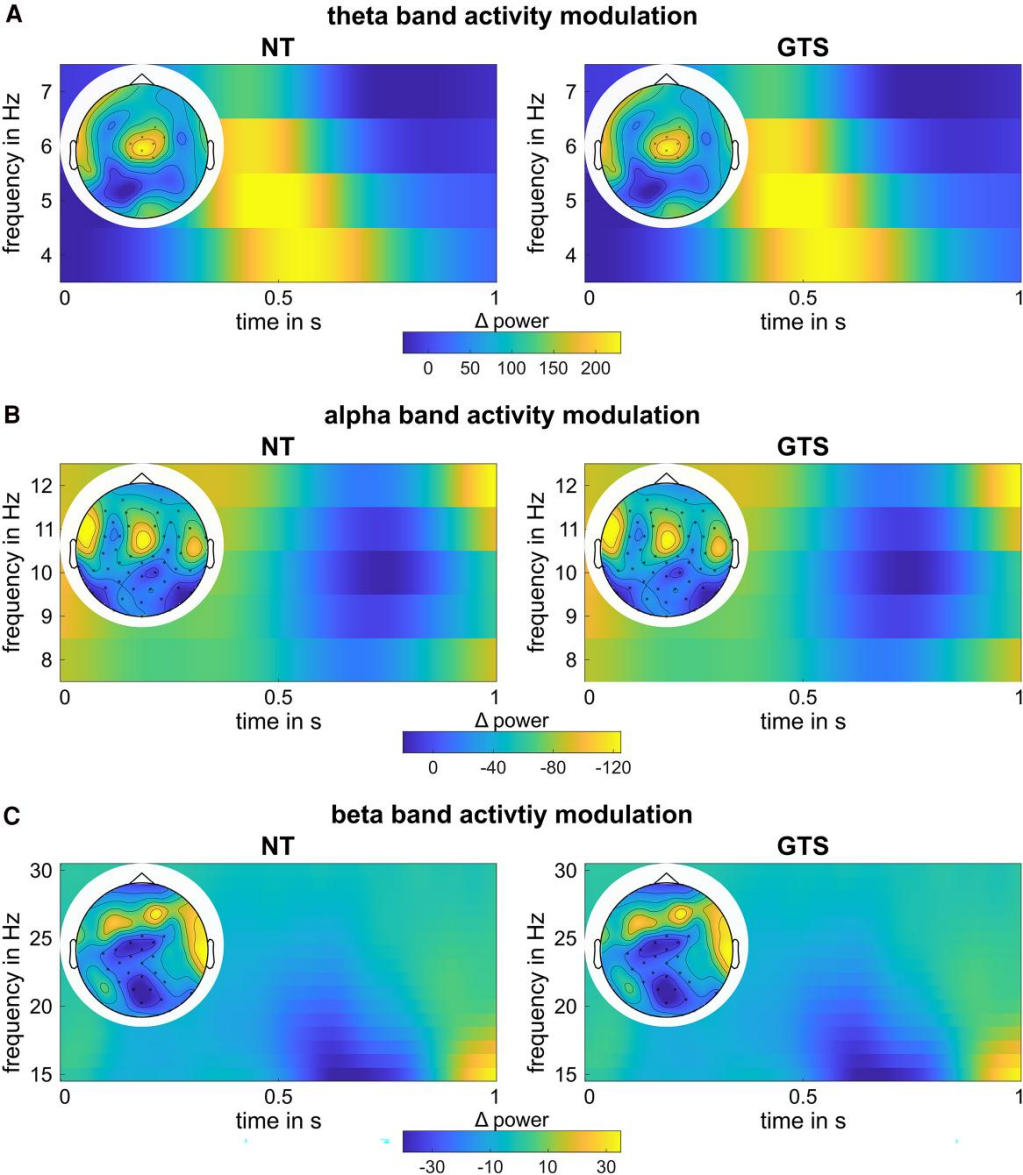
**Neurophysiological results at sensor level**. Time–frequency plots show the difference between overlapping and non-overlapping Nogo conditions at electrodes identified as significant in the cluster-based permutation test; time point zero reflects the presentation of the stimulus. Topographic plots highlight significant electrodes, averaged across time. Left side shows NT group (*N* = 30) results, right side shows GTS group (*N* = 30) results. (**A**) Results in the theta frequency band, showing a positive cluster in each NT (*T*_sum_ = 23.46, *P* = 0.026) and in GTS (*T*_sum_ = 23.46, *P* = 0.040). (**B**) Results in the alpha frequency band, showing a negative cluster in each NT (*T*_sum_ = −156.76, *P* = 0.002) and in GTS (*T*_sum_ = −156.76, *P* = 0.002). (**C**) Results in the beta frequency band, showing a negative cluster in each NT (*T*_sum_ = −61.46, *P* = 0.006) and in GTS (*T*_sum_ = −61.46, *P* = 0.002). Regarding between-group comparisons, no clusters were found through cluster-based permutation testing in all frequency bands.

The results of the cluster-based permutation testing on sensor level are shown in Fig. 3.

In the neurotypical group, a positive cluster of activity was found in the theta frequency band (*T*_sum_ = 23.46, *P* = 0.026; electrodes: Cz, FCz, FC1, CP1, FC2, CP2, F2, FC4), indicating higher theta band activity in the overlapping Nogo condition compared to the non-overlapping Nogo condition. In the alpha frequency band, cluster-based permutation testing revealed a negative cluster (*T*_sum_ = −156.76, *P* = 0.002) across almost all electrodes. Similarly, a negative cluster was found in the beta frequency band (*T*_sum_ = −61.46, *P* = 0.006) at central, frontal, parietal and occipital electrodes. In the GTS group, cluster-based permutation testing identified a positive cluster in the theta frequency band (*T*_sum_ = 23.46, *P* = 0.040; electrodes: Cz, FCz, FC1, CP1, FC2, CP2, F2, FC4). Additionally, a negative cluster was obtained in the alpha frequency band (*T*_sum_ = −156.76, *P* = 0.002) across almost all electrodes, and a similar negative cluster was observed in the beta frequency band (*T*_sum_ = −61.46, *P* = 0.002) at central, frontal, parietal and occipital electrodes. Regarding between-group comparisons, no clusters were found through cluster-based permutation testing for condition or overlap effect differences.

### Cluster-based permutation testing on source level

**Figure 4:**
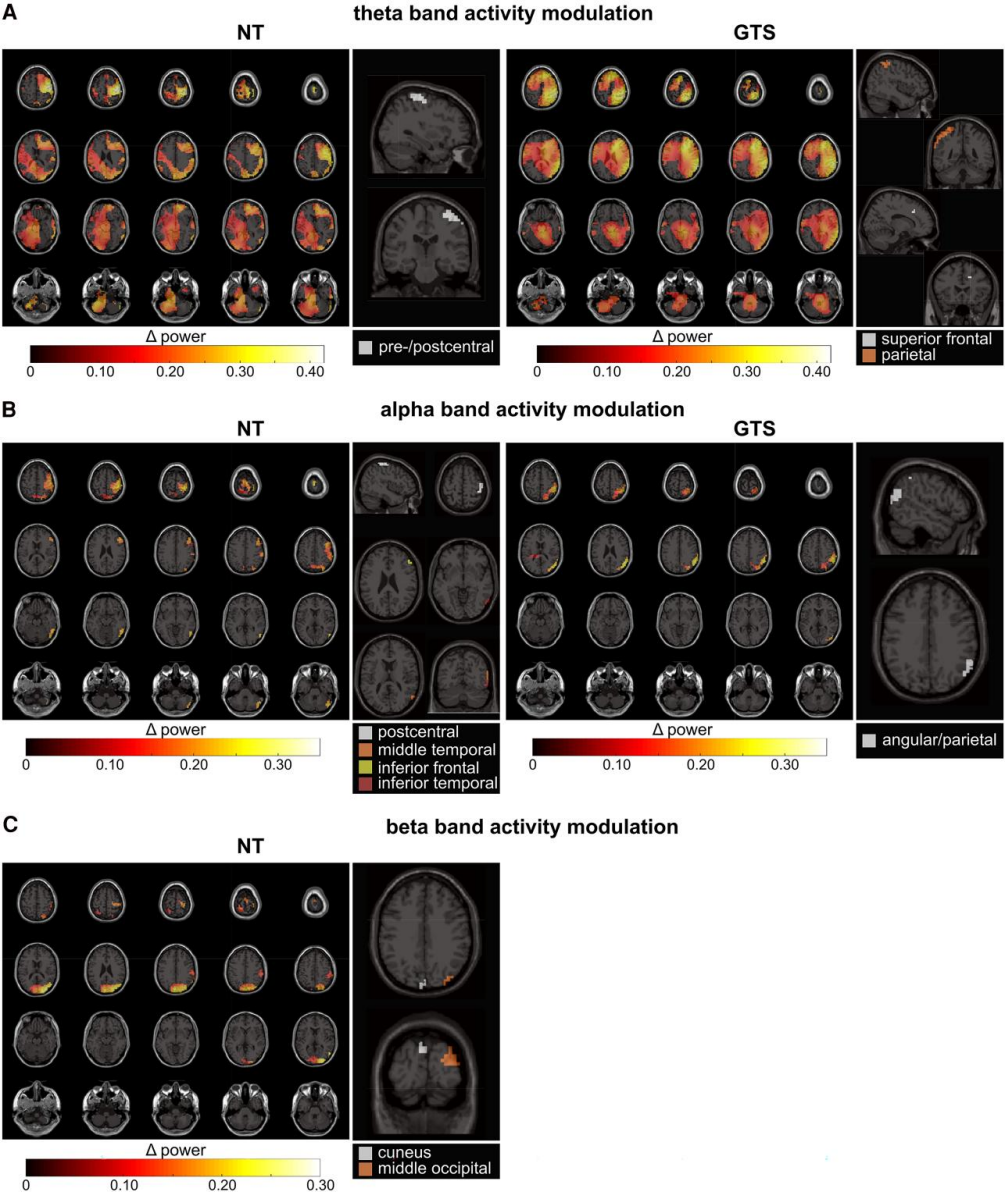
**Neurophysiological results at source level**. The left plots for each parcel show differences between overlapping and non-overlapping Nogo conditions in voxels identified as significant in cluster-based permutation testing. Colour scaling indicates the magnitude of power differences. The right plots highlight the top 1% of voxels with the largest differences. Left side shows NT group (*N* = 30) results, right side shows GTS group (*N* = 30) results. (**A**) Results in the theta frequency band, showing a positive cluster in each NT (*T*_sum_ = 27 896.72, *P* < 0.001) and in GTS (*T*_sum_ = 32 810.62, *P* < 0.001). (**B**) Results in the alpha frequency band, showing a positive cluster in each NT (*T*_sum_ = 4010.10, *P* = 0.019) and in GTS (*T*_sum_ = 2357.45, *P* = 0.040). (**C**) Results in the beta frequency band, showing a positive cluster in NT (*T*_sum_ = 3733.36, *P* = 0.034), but no significant cluster in GTS (*P* ≥ 0.176).

In the neurotypical group, a positive cluster for theta band activity was obtained by cluster-based permutation testing (*T*_sum_ = 27 896.72, *P* < 0.001). The top 1% difference could be localized in the right pre- and postcentral gyri. Further, there was a positive cluster in the alpha frequency band (*T*_sum_ = 4010.10, *P* = 0.019), with the highest difference localized in the right inferior temporal and occipital regions, right middle temporal cortex, right inferior frontal cortex and right postcentral gyrus. Also, in the beta frequency band, a positive cluster was revealed (*T*_sum_ = 3733.36, *P* = 0.034), and the highest difference was localized in the right occipital and middle temporal areas and in the left cuneus. The GTS group had a positive cluster in the theta frequency band (*T*_sum_ = 32 810.62, *P* < 0.001), localized in the right parietal and postcentral cortex and right superior frontal cortex. Moreover, a positive cluster in the alpha frequency band was obtained (*T*_sum_ = 2357.45, *P* = 0.040). The highest difference in the alpha frequency band was localized in the right angular, inferior parietal and middle temporal cortex. However, none of the clusters in the beta frequency band was significant (*P* ≥ 0.176). The results of the cluster-based permutation test, along with the regions of highest activity, are displayed in Fig. 4.

**Figure 5:**
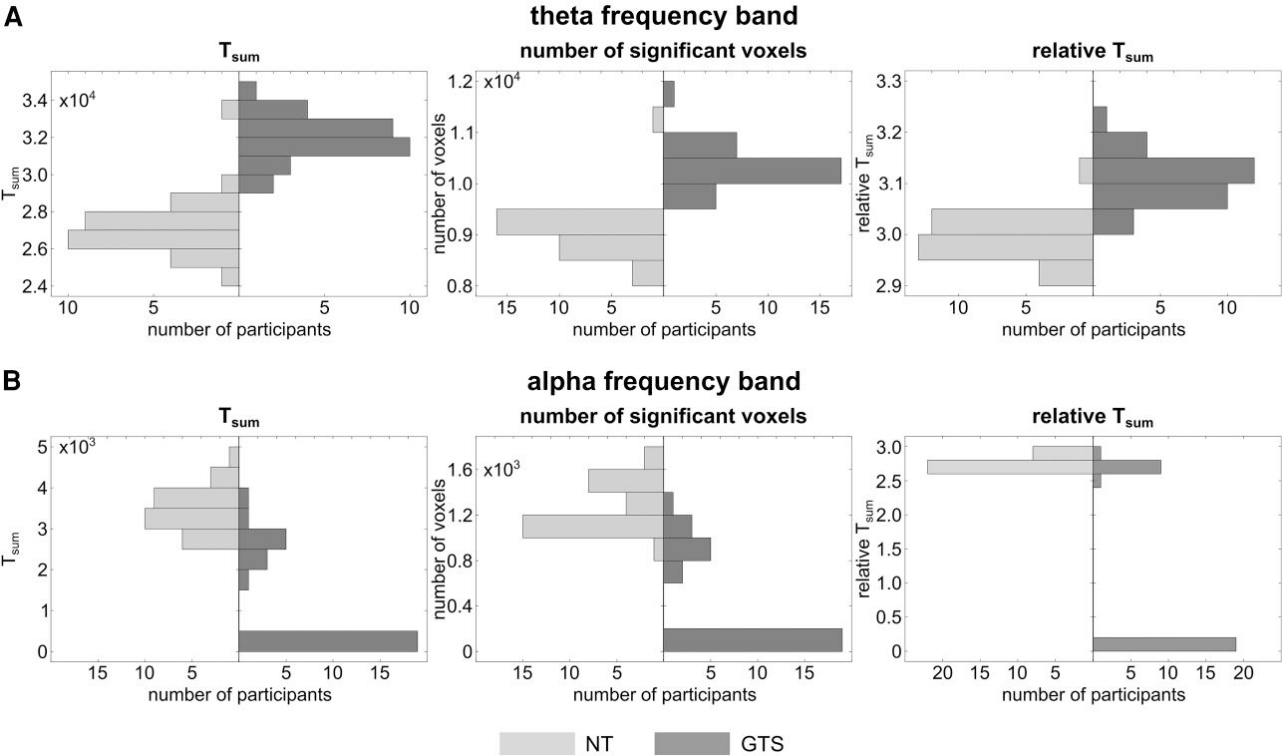
**Distribution of the cluster-based permutation test results**. Histograms show the distribution of outcome parameters from the leave-one-out approach of cluster-based permutation testing at source level. Parameters include the *T*_sum_ of the cluster-based permutation test, the number of significant voxels and the relative *T*_sum_ (*T*_sum_ normalized by the number of significant voxels; left to right). Results are displayed for NT (light grey; *N* = 30) and GTS (dark grey; *N* = 30). (**A**) Results for the theta frequency band, indicating a larger number of significant voxels (*Z* = −6.23, *P* < 0.001), a larger *T*_sum_ (*Z* = −6.24, *P* < 0.001) and a larger relative *T*_sum_ (*Z* = −6.33, *P* < 0.001) in the GTS group compared to the neurotypical individuals. (**B**) Results for the alpha frequency band, indicating a larger number of significant voxels (*Z* = −3.86, *P* < 0.001), a larger *T*_sum_ (*Z* = −4.03, *P* < 0.001) and a larger relative *T*_sum_ (*Z* = −3.68, *P* < 0.001) in the GTS group compared to the neurotypical individuals when treating the absence of significant clusters as missing value (NT: *N* = 30; GTS: *N* = 11). Also when assigning zeros in the case of absence of significant clusters (NT: *N* = 30; GTS: *N* = 30), there was a larger number of significant voxels (*Z* = −6.25, *P* < 0.001), a larger *T*_sum_ (*Z* = −6.34, *P* < 0.001) and a larger relative *T*_sum_ (*Z* = −6.16, *P* < 0.001) in the GTS group compared to the neurotypical individuals.

Comparing the results of the cluster-based permutation testing between the groups in the theta and the alpha frequency bands established significant group differences. With respect to the theta frequency band, all iterations yielded significant condition differences in both groups. The comparison of the results revealed a larger number of significant voxels (*Z* = −6.23, *P* < 0.001, *r* = −1.136), a larger *T*_sum_ (*Z* = −6.24, *P* < 0.001, *r* = −1.139) and a larger relative *T*_sum_ (*Z* = −6.33, *P* < 0.001, *r* = −1.155) in the GTS group (10 311 ± 391 voxels, *T*_sum_: 32 078.36 ± 1454.58, relative *T*_sum_: 3.11 ± 0.04) compared to the neurotypical individuals (9081 ± 457 voxels, *T*_sum_: 27 157.95 ± 1580.81, relative *T*_sum_: 2.99 ± 0.04). Since 19 of 30 iterations did not reveal significant clusters in the GTS group for the alpha frequency band, both approaches of dealing with the absence of significant clusters were followed. When the absence of significant clusters was set as a missing value, the neurotypical individuals (1278 ± 199 voxels, *T*_sum_: 3548.05 ± 583.62, relative *T*_sum_: 2.77 ± 0.04) had more significant voxels (*Z* = −3.86, *P* < 0.001, *r* = −0.704), a larger *T*_sum_ (*Z* = −4.03, *P* < 0.001, *r* = −0.736) and a larger relative *T*_sum_ (*Z* = −3.68, *P* < 0.001, *r* = −0.672) than the GTS group (980 ± 184 voxels, *T*_sum_: 2622.05 ± 464.69, relative *T*_sum_: 2.68 ± 0.09). When the absence of significant clusters was dealt with by assigning a zero, this only affected the values in the GTS group (359 ± 492 voxels, *T*_sum_: 961.42 ± 1313.80, relative *T*_sum_: 0.98 ± 1.31). However, the group difference remained significant (number of voxels: *Z* = −6.25, *P* < 0.001, *r* = −1.141; *T*_sum_: *Z* = −6.34, *P* < 0.001, *r* = −1.158; relative *T*_sum_: *Z* = −6.16, *P* < 0.001, *r* = −1.125). The distribution of values is presented in Fig. 5.

### Correlational analysis

None of the obtained correlation coefficients was significant given the Bonferroni-corrected alpha level of 0.001 (*ρ* ≤ 0.70, *P* ≥ 0.011). The complete results of the correlation analysis are presented in Supplementary Table 2.

## Discussion

The current study examined the relevance of oscillatory activity for perception–action integration processes in patients with GTS. The study was motivated by a novel concept according to which overly strong perception–action associations (bindings) are central for the understanding of GTS^3^ and by recent concepts according to which distinct processes during perception–action integration depend on theta, alpha and beta band activity.^13^ The findings reveal that individuals with GTS have increased perception–action association during response inhibition and that theta and alpha band activity modulations likely reflect a central mechanistic element in this.

The behavioural data (i.e. false alarm rates) show that response inhibition deteriorated in the experimental condition where features of the Nogo stimulus overlapped with features of the Go stimulus, replicating numerous previous findings.^47,48,54^ Importantly, this effect was stronger in individuals with GTS than neurotypical individuals. Thus, patients with GTS show difficulties when reconfiguration processes of stimulus–response associations are required during response inhibition. While this finding is not unprecedented,^9^ the current findings, for the first time, provide clear-cut evidence which neurophysiological processes likely underlie these peculiarities:

At the sensor level, both NT individuals and patients with GTS exhibited similar modulations in theta, alpha and beta band activity, with the findings for NT individuals specifically replicating previous results.^21,22,48,55^ However, differences between the two groups became evident when analysing the neurophysiological activity modulations at the source level: regarding beta band activity, only NT individuals revealed modulations between conditions requiring reconfiguring perception–action codes (i.e. overlapping Nogo trials) and those that did not (i.e. non-overlapping Nogo trials). In NT individuals, beta band modulations were most prominent in secondary visual association areas within the ventral visual stream (see Fig. 4, DBSCAN results). Visual features define overlapping and non-overlapping Nogo trials, and handling perception–action codes requires that information about what specific features are present is processed.^56^ Moreover, a reconfiguration of bound perception–action codes has been linked to beta band activity.^57,58^ Neurotypical individuals, in contrast to patients with GTS, seem to modulate beta band activity to enable the dynamic handling of perception–action codes. This may contribute to the observed abnormalities in patients with GTS; however, this dissociation has to be interpreted cautiously, as the lack of significant modulatory effects in GTS could have other explanations beyond the absence of an effect. More importantly, modulations in theta and alpha band activity between the contrasted Nogo conditions demonstrated differential modulation patterns of NT individuals and patients with GTS (see Figs. 4 and 5). Patients with GTS revealed stronger and more extended cortical activity modulations between overlapping and non-overlapping Nogo trials in the theta band compared to NT individuals. Within the alpha band, the pattern was reversed: patients with GTS had weaker and more restricted cortical activity modulations between overlapping and non-overlapping Nogo trials compared to NT individuals. This pattern of effects was consistent across different approaches to quantify the group differences in cortical activity modulations, underlining the robustness of the differential modulation. Stronger bindings between perception–action codes in patients with GTS are thus a function of changes in theta and alpha band activity. This puts theta and alpha band activity modulations in the centre of pathophysiological processes in GTS, particularly regarding response inhibition. The increased theta band activity in patients with GTS compared to NT individuals likely reflects a stronger association of perception–action codes and likely higher costs to reconfigure perception–action codes during overlapping Nogo trials. The centre of activity modulations (see Fig. 4, DBSCAN results) was in the middle frontal and superior parietal regions. Theta activity in these areas can be associated with input integration and linking stimuli and responses.^21,22,59^ The activity modulation related to these processes is increased in patients with GTS. Importantly, these processes may not be sufficiently modulated or counteracted by other forms of neural activity. Previous findings suggest that the processes reflected in theta band activity are controlled by functions of the alpha frequency band in a top-down modulating manner.^13,22^ In fact, the modulation peak of alpha band activity occurs after the modulation peak of theta band activity, suggesting that alpha band-related processes might affect the duration or the modulation amplitude of theta band-related processes.^22^ More specifically, alpha band modulation was stronger and broader in NT individuals than in patients with GTS. Thus, NT individuals, also showing better behavioural performance particularly in overlapping Nogo trials, probably more efficiently adapt processes modulating theta band activity during perception–action integration.^13,22^ Intriguingly, neither behavioural performance nor neurophysiological activity modulations showed substantial correlations with tic-related disease parameters. This suggests that the observed alterations in behavioural performance and neurophysiological modulations are not directly linked to tics but instead constitute a novel aspect of Tourette's syndrome. Future studies should further examine the influence of tic-related medication and comorbidities, as the sample size in the current study precluded meaningful analyses on this aspect. Additionally, longitudinal approaches should investigate whether neurophysiological alterations precede the onset of symptoms or emerge only after a certain duration, to clarify the temporal relationship between neurophysiological changes and symptomatology.

Our findings suggest that patients with GTS exhibit an exaggerated association between perception–action codes, reflected by increased theta band activity modulation, which is less controlled by other forms of neural activity, particularly alpha band activity, when dynamic management of perception–action codes is required, i.e. during the reconfiguration of perception–action codes. This pattern strongly suggests that theta^14-16^ and alpha band activity^20^ likely play a role in the pathophysiology of GTS and relate both forms of activity to each other. The robust differential modulation suggests that a mechanistic principle underlying GTS pathology has been identified in this study, which is clinically relevant. Mechanisms underlying perception–action integration have been discussed as central for cognitive-behavioural interventions in GTS.^11^ This is because such interventions rely on reconfiguring perception–action associations.^60-62^

To summarize, our study provides novel insights into perception–action integration in patients with GTS, emphasizing the mechanistic role of theta and alpha band activity. We demonstrated that GTS is characterized by exaggerated perception–action associations during inhibitory control reflected in altered neural oscillatory patterns. Specifically, modulation of theta band activity was heightened, suggesting a stronger, more rigid association between perception and action codes, while alpha-band modulations were weaker, indicating insufficient control over these processes. This differential modulation highlights a central pathophysiological feature in GTS, elucidating why response inhibition is impaired during tasks requiring dynamic reconfiguration perception–action codes. These findings advance our understanding of GTS mechanisms and have important implications for targeting cognitive-behavioural interventions.

## Supplementary Material

fcaf172_Supplementary_Data

## Data Availability

The data that support the findings of this study are available from the corresponding author, upon reasonable request. The code used to conduct the described analyses can be obtained in an OSF repository: https://osf.io/yx4cw/?view_only=e784507f509d4dc39fe4171c5c31f0d5.
