# Supplementary material for: Differential modulation of neural oscillations in perception-action links in Tourette syndrome
Source: Brain Commun. 2025 May 5;7(3):fcaf172. doi: 10.1093/braincomms/fcaf172 (PMC12070268; doi:10.1093/braincomms/fcaf172)
Supplement: fcaf172_Supplementary_Data [file fcaf172_supplementary_data.docx]

**Supplementary Material**

**Differential Modulation of Neural Oscillations in Perception-Action Links in Tourette Syndrome**

Astrid Prochnow, Annet Bluschke, Tina Rawish, Julia Friedrich, Yifan Hao, Christian Frings, Tobias Bäumer, Alexander Münchau, Christian Beste

**Supplementary Analysis: Association of alpha and theta band modulations**

***Methods***

Time-frequency analysis was performed using a wavelet transform approach in FieldTrip (Oostenveld et al., 2010). For the segmented EEG data for each subject, time-frequency decomposition was conducted using Morlet wavelets. Frequencies of interest ranged from 3 to 30 Hz, with the time axis preserved in full. Padding was applied to ensure sufficient frequency resolution. Power spectra were computed while retaining individual trials.

To investigate condition-related effects using a data-driven electrode selection approach, electrodes showing significant differences between the overlapping and non-overlapping conditions in the cluster-based permutation tests at the sensor level were included for subsequent analysis. For each subject, time-frequency data from these electrodes were extracted and averaged across electrodes, time (0–1 s post-stimulus), and frequency band (theta: 4–7 Hz; alpha: 8–12 Hz). The resulting data were organized by subject, group, condition, and trial. A linear mixed-effects model was then applied to examine interactions between theta and alpha band activity, group, and condition, incorporating a random intercept to account for inter-subject variability.

In addition, data from the non-overlapping condition were subtracted from the overlapping condition at the electrodes showing significant differences between the overlapping and non-overlapping conditions in the cluster-based permutation tests at the sensor level to obtain a differential power value for each subject. For each subject, resulting differences from these electrodes were extracted and averaged across electrodes, time (0–1 s post-stimulus), and frequency band (theta: 4–7 Hz; alpha: 8–12 Hz). The resulting data were organized by subject and group. A linear mixed-effects model was applied to examine interactions between theta and alpha band activity and group while accounting for inter-subject variability.

***Results***

*Theta band activity as outcome*

The linear regression model with the predictors alpha band activity, condition, and group and the outcome theta band activity was fitted using maximum likelihood estimation (ML), based on 8,802 observations (R² = .22; adjusted R² = .22). The intercept was significant (*b* = 271.3, *SE* = 49.56, *t*(8794) = 5.47, *p* < .001). The main effects of condition (*b* = 122.7, *SE* = 16.52, *t*(8794) = 7.43, *p* < .001) and alpha band activity (*b* = .22, *SE* = .05, *t*(8794) = 4.35, *p* < .001) were significant, while none of the other predictors nor their interactions significantly predicted theta band activity (all p ≥ .147).

The linear regression model with the predictors alpha band modulation and group and the outcome theta band modulation was fitted using maximum likelihood estimation (ML), based on 60 observations (R² = .57; adjusted R² = .54). The intercept was significant (*b* = 115.99, *SE* = 27.08, *t*(56) = 4.28, *p* < .001). None of the predictors significantly influenced the outcome (all p ≥ .083).

*Alpha band activity as outcome*

The linear regression model with the predictors theta band activity, condition, and group and the outcome alpha band activity was fitted using maximum likelihood estimation (ML), based on 8,802 observations (R² = .56; adjusted R² = .56). The intercept was significant (*b* = 278.39, *SE* = 73.95, *t*(8794) = 3.76, *p* < .001). The main effect of theta band activity (*b* = .21, *SE* = .04, *t*(8794) = 5.56, *p* < .001), the interaction of theta band activity and group (*b* = .12, *SE* = .05, *t*(8794) = 2.18, *p* = .029), and the interaction of theta band activity and condition (*b* = -.08, *SE* = .02, *t*(8794) = -3.76, *p* < .001) were significant, while none of the other predictors nor their interactions significantly predicted theta band activity (all p ≥ .098).

The linear regression model with the predictors theta band modulation and group and the outcome alpha band modulation was fitted using maximum likelihood estimation (ML), based on 60 observations (R² = .59; adjusted R² = .56). The intercept was significant (*b* = -58.24, *SE* = 21.76, *t*(56) = -2.68, *p* = .010). Theta band modulation was a significant predictor of alpha band modulation (*b* = .32, *SE* = .14, *t*(56) = 2.26, *p* = .028), however, neither group nor the interaction of group and theta band modulation had a significant effect (all p ≥ .194).

***References***

Oostenveld, R., Fries, P., Maris, E., & Schoffelen, J.-M. (2010). FieldTrip: Open Source Software for Advanced Analysis of MEG, EEG, and Invasive Electrophysiological Data. *Computational Intelligence and Neuroscience*, *2011*, e156869. https://doi.org/10.1155/2011/156869

**Supplementary Table 1 Clinical characteristics of the patients with GTS included in the study.**

| **Patient** | **Age** | **Sex** | **Disease Duration (Years)** | **DCI (0–100)** | **YGTSS Total (0–100)** | **YGTSS Tics (0–50)** | **YBOCS (0–40)** | **Medication** |
| --- | --- | --- | --- | --- | --- | --- | --- | --- |
| 1 | 19 | Male | 10 | 83 | 31 | 31 | 10 |  |
| 2 | 20 | Female | 14 | 93 | 50 | 30 | 14 |  |
| 3 | 29 | Female | 15 | 47 | 34 | 14 | 0 | Bedrocan |
| 4 | 29 | Female | 22 | 64 | 15 | 15 | 0 | Aripiprazol, Nabiximols |
| 5 | 20 | Male | 12 | 49 | 20 | 10 | 0 |  |
| 6 | 24 | Female | 11 | 36 | 40 | 10 | 0 |  |
| 7 | 36 | Male | 30 | 64 | 50 | 30 | 0 |  |
| 8 | 36 | Male | 32 | 100 | 53 | 33 | 2 |  |
| 9 | 21 | Female | 12 | 53 | 33 | 23 | 2 | Aripirazol |
| 10 | 23 | Female | 17 | 84 | 51 | 31 | 8 |  |
| 11 | 24 | Male | 14 | 45 | 32 | 22 | 0 |  |
| 12 | 28 | Male | 22 | 43 | 24 | 14 | 19 | Aripripazol |
| 13 | 35 | Male | 29 | 75 | 48 | 28 | 8 |  |
| 14 | 19 | Male | 9 | 77 | 29 | 9 | 0 |  |
| 15 | 19 | Male | 15 | 59 | 60 | 30 | 0 |  |
| 16 | 31 | Female | 27 | 100 | 78 | 38 | 19 |  |
| 17 | 28 | Female | 24 | 75 | 56 | 26 | 0 | Aripiprazol |
| 18 | 20 | Female | 14 | 42 | 26 | 16 | 0 |  |
| 19 | 21 | Female | 16 | 46 | 56 | 16 | 0 | Nabiximols |
| 20 | 40 | Female | 36 | 65 | 60 | 30 | 0 |  |
| 21 | 35 | Male | 17 | 62 | 65 | 35 | 20 | Sulpirid |
| 22 | 18 | Male | 7 | 61 | 53 | 23 | 6 |  |
| 23 | 22 | Male | 16 | 44 | 25 | 15 | 11 |  |
| 24 | 35 | Male | 29 | 50 | 47 | 27 | 8 |  |
| 25 | 29 | Male | 23 | 88 | 28 | 18 | 0 | Amisulprid |
| 26 | 26 | Male | 20 | 61 | 56 | 26 | 8 | Pimozid |
| 27 | 24 | Female | 17 | 80 | 51 | 21 | 15 | Aripiprazol |
| 28 | 21 | Male | 15 | 54 | 15 | 15 | 6 |  |
| 29 | 40 | Male | 25 | 38 | 31 | 11 | 7 |  |
| 30 | 25 | Male | 20 | 68 | 41 | 31 | 5 | Aripriprazol |

DCI = Diagnostic Confidence Index; YBOCS = Yale Brown Obsessive Compulsive Scale; YGTSS = Yale Global Tic Severity Scale.

**Supplementary Table 2 Spearman’s correlation coefficients and corresponding uncorrected p-values.**

|  | overlap effect behavior | alpha Tsum | alpha number of voxels | alpha relative Tsum | theta Tsum | theta number of voxels | theta relative Tsum | duration of disease | YGTSS motor | YGTSS vocal | YGTSS total | GTS DCI | YBOCS |
| --- | --- | --- | --- | --- | --- | --- | --- | --- | --- | --- | --- | --- | --- |
| overlap effect behavior | -- | *p* = .016 | *p* = .018 | *p* = .011 | *p* = .071 | *p* = .057 | *p* = .496 | *p* = .013 | *p* = .829 | *p* = .662 | *p* = .321 | *p* = .499 | *p* = .622 |
| alpha T_sum_ | ρ = .435 | -- | *p* < .001 | *p* < .001 | *p* = .117 | *p* = .038 | *p* = .545 | *p* = .681 | *p* = .239 | *p* = .271 | *p* = .394 | *p* = .056 | *p* = .760 |
| alpha number of voxels | ρ = .428 | ρ = .996 | -- | *p* < .001 | *p* = .116 | *p* = .034 | *p* = .518 | *p* = .611 | *p* = .270 | *p* = .358 | *p* = .442 | *p* = .077 | *p* = .684 |
| alpha relative T_sum_ | ρ = .459 | ρ = .913 | ρ = .903 | -- | *p* = .045 | *p* = .020 | *p* = .909 | *p* = .598 | *p* = .495 | *p* = .190 | *p* = .423 | *p* = .128 | *p* = .816 |
| theta T_sum_ | ρ = .334 | ρ = .292 | ρ = .293 | ρ = .368 | -- | *p* < .001 | *p* = .001 | *p* = .327 | *p* = .707 | *p* = .594 | *p* = .551 | *p* = .248 | *p* = .826 |
| theta number of voxels | ρ = .352 | ρ = .381 | ρ = .388 | ρ = .422 | ρ = .949 | -- | *p* = .081 | *p* = .303 | *p* = .603 | p = .612 | *p* = .367 | *p* = .279 | *p* = .765 |
| theta relative T_sum_ | ρ = .129 | ρ = -.115 | ρ = -.123 | ρ = .022 | ρ = .560 | ρ = .324 | -- | *p* = .800 | *p* = .963 | *p* = .648 | *p* = .753 | *p* = .243 | *p* = .437 |
| duration of disease | ρ = -.449 | ρ = -.078 | ρ = -.097 | ρ = -.100 | ρ = -.185 | - ρ = .195 | ρ = -.048 | -- | *p* = .131 | *p* = .040 | *p* = .093 | *p* = .138 | *p* = .511 |
| YGTSS motor | ρ = .041 | ρ = .222 | ρ = .208 | ρ = .130 | ρ = .072 | ρ = .099 | ρ = .009 | ρ = .282 | -- | *p* = .027 | *p* < .001 | *p* < .001 | *p* = .495 |
| YGTSS vocal | ρ = .083 | ρ = .207 | ρ = .174 | ρ = .246 | ρ = .101 | ρ = .096 | ρ = .087 | ρ = .377 | ρ = .403 | -- | *p* = .001 | *p* = .004 | *p* = .010 |
| YGTSS total | ρ = -.187 | ρ = .161 | ρ = .146 | ρ = .152 | ρ = .113 | ρ = .171 | ρ = -.060 | ρ = .312 | ρ = .619 | ρ = .588 | -- | *p* = .038 | *p* = .381 |
| GTS DCI | ρ = .128 | ρ = .353 | ρ = .328 | ρ = .284 | ρ = .217 | ρ = .204 | ρ = .220 | ρ = .278 | ρ = .611 | ρ = .508 | ρ = .381 | -- | *p* = .223 |
| YBOCS | ρ = .094 | ρ = -.058 | ρ = -.077 | ρ = -.044 | ρ = .042 | ρ = .057 | ρ = .147 | ρ = .125 | ρ = .129 | ρ = .464 | ρ = .166 | ρ = .229 | -- |

Above the diagonal, p-values are presented, while below the diagonal, the respective Spearman’s correlation coefficients ρ are shown. All behavioral measures and clinical reports have been averaged using a leave-one-out approach in order to be comparable with the neurophysiological effects. Numbers presented in grey font indicate correlations that are not of interest and were therefore excluded from the Bonferroni correction. DCI = Diagnostic Confidence Index; YBOCS = Yale Brown Obsessive Compulsive Scale; YGTSS = Yale Global Tic Severity Scale.
